# Structured feedback and operative video debriefing with critical view of safety annotation in training of laparoscopic cholecystectomy: a randomized controlled study

**DOI:** 10.1007/s00464-024-10843-6

**Published:** 2024-04-23

**Authors:** Amila Cizmic, Frida Häberle, Philipp A. Wise, Felix Müller, Felix Gabel, Pietro Mascagni, Babak Namazi, Martin Wagner, Daniel A. Hashimoto, Amin Madani, Adnan Alseidi, Thilo Hackert, Beat P. Müller-Stich, Felix Nickel

**Affiliations:** 1 Department of General, Visceral and Thoracic Surgery, University Medical Center Hamburg-Eppendorf, Martinistraße 52, 20251 Hamburg, Germany; 2 Department of General, Visceral and Transplantation Surgery, Heidelberg University Hospital, Heidelberg, Germany; 3 Fondazione Policlinico Universitario Agostino Gemelli IRCCS, Rome, Italy; 4 Institute of Image-Guided Surgery, IHU-Strasbourg, Strasbourg, France; 5 Center for Evidence-Based Simulation, Baylor University Medical Center, Dallas, USA; 6 Department of Visceral, Thoracic and Vascular Surgery, Faculty of Medicine and University Hospital Carl Gustav Carus, Technische Universität Dresden, Dresden, Germany; 7 Penn Computer Assisted Surgery and Outcomes (PCASO) Laboratory, Department of Surgery, Department of Computer and Information Science, University of Pennsylvania, Philadelphia, USA; 8 Surgical Artificial Intelligence Research Academy (SARA), Department of Surgery, University Health Network, Toronto, Canada; 9 Department of Surgery, University of California - San Francisco, San Francisco, USA; 10 Department of Surgery, Clarunis – University Centre for Gastrointestinal and Liver Diseases, Basel, Switzerland; 11 HIDSS4Health – Helmholtz Information and Data Science School for Health, Karlsruhe, Heidelberg, Germany

**Keywords:** Minimally invasive surgery, Training, Laparoscopic cholecystectomy, Video debriefing, Structured feedback, CVS annotation

## Abstract

**Background:**

The learning curve in minimally invasive surgery (MIS) is lengthened compared to open surgery. It has been reported that structured feedback and training in teams of two trainees improves MIS training and MIS performance. Annotation of surgical images and videos may prove beneficial for surgical training. This study investigated whether structured feedback and video debriefing, including annotation of critical view of safety (CVS), have beneficial learning effects in a predefined, multi-modal MIS training curriculum in teams of two trainees.

**Methods:**

This randomized-controlled single-center study included medical students without MIS experience (*n* = 80). The participants first completed a standardized and structured multi-modal MIS training curriculum. They were then randomly divided into two groups (*n* = 40 each), and four laparoscopic cholecystectomies (LCs) were performed on ex-vivo porcine livers each. Students in the intervention group received structured feedback after each LC, consisting of LC performance evaluations through tutor-trainee joint video debriefing and CVS video annotation. Performance was evaluated using global and LC-specific Objective Structured Assessments of Technical Skills (OSATS) and Global Operative Assessment of Laparoscopic Skills (GOALS) scores.

**Results:**

The participants in the intervention group had higher global and LC-specific OSATS as well as global and LC-specific GOALS scores than the participants in the control group (25.5 ± 7.3 vs. 23.4 ± 5.1, *p* = 0.003; 47.6 ± 12.9 vs. 36 ± 12.8, *p* < 0.001; 17.5 ± 4.4 vs. 16 ± 3.8, *p* < 0.001; 6.6 ± 2.3 vs. 5.9 ± 2.1, *p* = 0.005). The intervention group achieved CVS more often than the control group (1. LC: 20 vs. 10 participants, *p* = 0.037, 2. LC: 24 vs. 8, *p* = 0.001, 3. LC: 31 vs. 8, *p* < 0.001, 4. LC: 31 vs. 10, *p* < 0.001).

**Conclusions:**

Structured feedback and video debriefing with CVS annotation improves CVS achievement and ex-vivo porcine LC training performance based on OSATS and GOALS scores.

Minimally invasive surgery (MIS) has significant advantages for patients compared to open surgery [1], including shorter postoperative patient recovery, decreased perioperative complications, smaller incisions, and less blood loss [2, 3]. However, the surgical skills required to perform image-guided surgery differ from those of traditional open surgery, requiring different training methods [4–8]. This might represent a challenge for novice surgeons whose learning curve is influenced by factors such as a limited 2-dimensional view, challenging hand–eye coordination, difficult instrument handling, and inhibited haptic response [9].

Many MIS training modalities have proven to positively affect the development of MIS skills in a patient-safe environment [6, 7, 9–16]. Simulation is a complementary training modality that helps acquire surgical abilities, accelerating the learning curve in a controlled, safe, and standardized environment [10, 17–19]. The main goal of simulation training is to acquire and improve surgical skills and then transfer these skills into the operating room (OR) [6, 20]. One of the MIS simulation training aspects is to provide structured feedback to the trainees after an MIS training session [21]. Feedback is an educational technique and a social interaction between tutor and trainee in a respectful and trusting relationship [22]. In MIS training, individual feedback has proven to positively impact the performance and confidence of the trainees [21, 23, 24]. Video debriefing of the performed surgical procedure as a form of feedback improves surgical training and the certitude of the trainees [23, 25–27]. Video debriefing has been used in a randomized-controlled setting in laparoscopic cholecystectomy (LC) on Virtual Reality (VR) trainer in laparoscopic novices and showed significant improvement of laparoscopic skills [27]. However, the video debriefing in this study was performed only after VR LCs, and it did not include the annotation of the critical view of safety (CVS) or the identification of the safe (Go) and dangerous (No-Go) zones of dissection during LC. Timing out to assess the CVS during LC has been shown to increase the rate of intraoperative CVS achievement, which may lead to a reduction of complications [28]. Identifying Go and No-Go zones of dissection during LC using artificial intelligence (AI) has been used as a form of intraoperative guidance and can potentially reduce intraoperative mistakes [29]. These modalities thus bear the potential to be used in MIS training as part of structured feedback and video debriefing after LC to improve the performance in LC.

This study aimed to assess the effects of structured feedback with video debriefing on training success in trainees undergoing a predefined multi-modal MIS training. The MIS training curriculum was completed in teams of two trainees and included repetitive ex-vivo porcine LCs.

## Materials and methods

### Study design

According to the CONSORT guidelines for randomized-controlled trials, this study was designed as a prospective, single-center, two-arm, randomized-controlled study [30]. The primary aim of the study was to investigate whether there is a benefit of structured feedback and video debriefing with CVS annotation after LCs in teams of two trainees compared to training without structured feedback and video debriefing.

### Study setting and participants

The study was conducted between September 2021 and December 2022 at the MIS training center at the Department of General, Visceral, and Transplantation Surgery at the University Hospital Heidelberg, Germany. The MIS training center offers voluntary MIS training courses to medical students at Heidelberg University during their clinical years. The local ethics committee gave its approval (S-436/2018).

### Inclusion and exclusion criteria

Inclusion criteria were medical students enrolled at Heidelberg University Medical School during their clinical years. The exclusion criteria were participation in previous MIS training courses or experience in MIS.

### Study flow

**Fig. 1:**
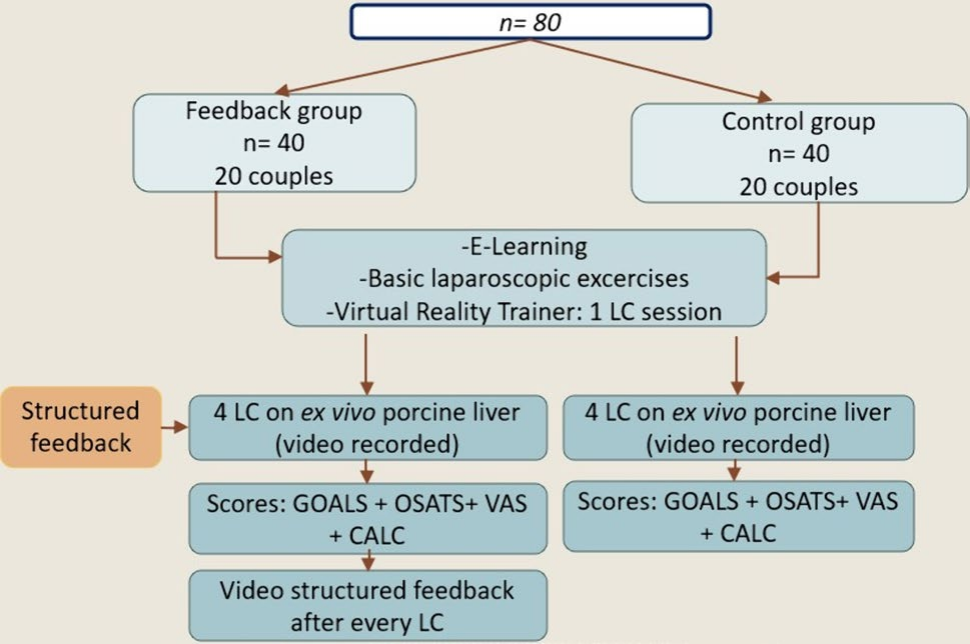
Flowchart of the study. *LC* laparoscopic cholecystectomy; *OSATS* Objective Structured Assessment of Technical Skills; *GOALS* Global Operative Assessment of Laparoscopic Skills; *VAS* visual assessment score for LC difficulty; *CALC* complication assessment score of LC

**Fig. 2:**
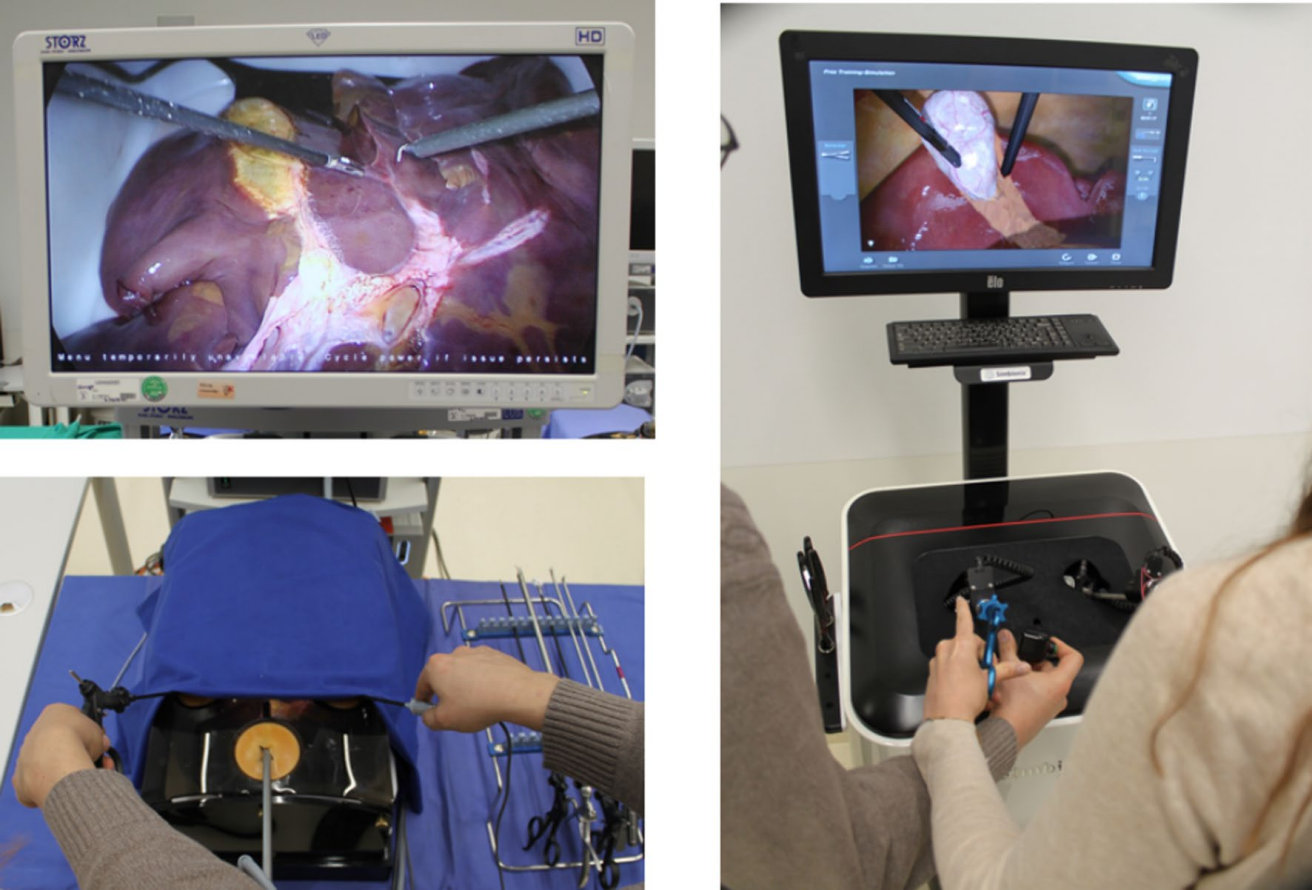
Training setting for laparoscopic cholecystectomy on an ex-vivo porcine liver (on the left) and on the Virtual Reality trainer (on the right)

Both groups underwent a structured MIS basic training consisting of 2 hours of e-learning about the basic MIS skills and LC (http://www.webop.de, http://www.websurg.com) under the supervision of a trained tutor [31, 32] (Fig. 1). Afterward, the trainees performed 6 hours of practical basic MIS training on a Szabo–Berci–Sackier Box Trainer and a standard laparoscopy tower (KARL STORZ GmbH & Co. KG, Tuttlingen, Germany). The exercises included: laparoscopic camera guidance, clamping six rubber bands in a device with six screws, pulling a rubber band into a device made of eyelets and hooks, cutting a predefined circle on a paper sheet, needle-guidance, and continuous and interrupted suturing on a 3D printed wound model. The basic MIS training encompassed a 2 hours basic module and LC on a VR trainer (Simbionix LAP Mentor) (Fig. 2, on the right).

After the MIS basic training, the two groups performed 4 LCs in teams of two (Fig. 2, on the left). A trained tutor provided both groups with assistance and verbal guidance during each LC on demand. The tutor was previously trained by an experienced board-certified surgeon in performing and teaching LC on ex-vivo porcine livers.

The students in the control group performed the four LCs without structured feedback, video debriefing, and CVS annotation, whereas the intervention group students were provided with structured feedback and video debriefing with CVS annotation as a form of intervention. All ex-vivo porcine LCs were captured on video and evaluated using the standardized, validated assessment systems (OSATS and GOALS scores) on-site for structured feedback and by a blinded rater for outcome assessment [33, 34].

### Intervention

**Fig. 3:**
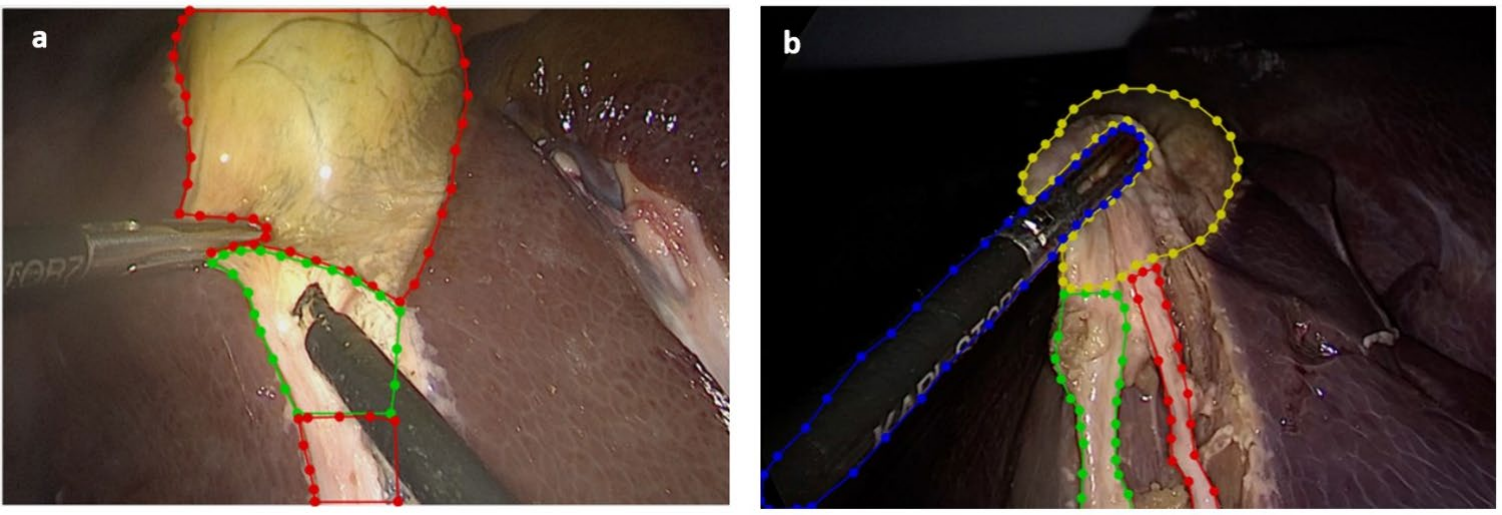
**a** Annotation of Go- (marked in green) and No-Go zones (marked in red) using open-access software LabelMe; **b** Annotations of the important anatomical structures and achievement of the CVS: Gallbladder (yellow), cystic artery (red), cystic duct (green), instrument (blue)

After each LC, the feedback group received structured feedback and video debriefing with CVS annotation from a trained tutor. The post-LC structured feedback and video debriefing consisted of a joined video assessment of the performed LC reflecting on important steps, complications, and potential improvement of the performance of the procedure (exposure, instrument holding, dissection, clip positioning, cutting). Video debriefing consisted of annotating four predefined video frames on an open-source platform using the annotation tool LabelMe [35]. The four predefined video frames were: starting point of the LC after instrument insertion, first cauterization, identification of the cystic duct and artery, and 10s before clipping. The main goal of CVS annotations was to provide visual guidelines to perform a safe LC. The tutor and the trainee annotated the Go- and No-Go zones in the first two video frames. The Go- and No-Go zones were defined as safe (Go) and dangerous (No-Go) zones of dissection (Fig. 3a) [29].

Video frames 3 and 4 were annotated by the tutor and the trainee regarding the identification of the CVS (Fig. 3b) [36].

### Primary outcomes

The study's primary outcome was comparing the LC technical skills of two groups using global and task-specific OSATS and GOALS assessment scores after each LC performed [34, 37].

OSATS is a standardized, validated assessment tool implemented in many academic centers to measure operative performance [34]. The global OSATS score evaluates tissue respect, efficiency, usage, and knowledge of the instruments, camera assistance, and workflow (35 in total attainable points). The task-specific OSATS score assesses the following LC aspects: (a) retraction of the gallbladder, (b) the Calot’s triangle preparation, (c) the preparation of the cystic duct, (d) the preparation of the cystic artery, (e) the preparation of the gallbladder, (f) the clipping and cutting, (g) the knowledge of the procedure, and (h) the end product’s quality (70 attainable points). GOALS can be used as a general performance assessment tool for any MIS procedure and procedure-specific assessment, such as video assessment of LC in the learning stage observed in medical students [37].

### Secondary outcomes

The total time spent performing the LC, the complication assessment, CVS achievement, and difficulty of the LC were recorded as well. Complications such as perforations, injuries on the cystic artery and/or cystic duct, and misplacement of the clips were assessed using a 3-point Likert scale [38]. The difficulty of each LC was evaluated by the trained tutor of the MIS section supervising the trainees using the visual analog scale (VAS) [37].

Various data regarding the subjective effects of the predefined training were recorded for each trainee using predefined questionaries. The questions were related to the subjective assessment of personal improvement in performing the LC and acquiring MIS skills. The feedback group received additional questions associated with the feedback and video debriefing.

### Sample size

The sample size calculation was based on a previous study in the same setting [39]. With a two-sided *α* = 0.05, the sample size gives 80% power to detect a standardized effect of *d* = 0.64 with a power of 80%. This effect represents approximately 2.5 points for the general skills scale and about 3.5 points for the specific skills scale. As the general skills scale parameters range from 1 to 5 and those for the specific skills from 2 to 10, the effect would reflect an improvement of precisely one scale unit, which is reasonably small. The sample size determination for the total scores of general and specific scales can only be estimated as the correlation between the two scales is unknown. Assuming a positive correlation of *ρ* = 0.5, the standard deviation for the total scores of the scales would be 7.86 for both groups. With the sample size of 40 participants per group and *α* = 0.05 two-tailed, a difference of 5 points would be detected (for example, 3 points for the general skills area and 2 points for the specific skills area) with a power of 80%.

### Statistical analysis

Statistical analysis and descriptive statistics were performed with the SPSS software (version 25.0, IBM SPSS Inc., Chicago, Illinois, USA), and data were given as absolute frequency and mean ± standard deviation. Differences between the LC were assessed using the *t*-Test for independent samples in parametric data and the Mann–Whitney *U* test for independent samples in the case of non-parametric data. For binary endpoints, group differences were calculated using the Chi-square test. A *p*-value of *p* < 0.05 was considered statistically significant.

## Results

Eighty medical students were included in the study. The medical students were randomized into the feedback group (*n* = 40) and the control group (*n* = 40). All 80 trainees completed the study at the Department of General, Visceral and Transplantation Surgery, Heidelberg University Hospital, Germany.

### Primary outcomes

The total global and task-specific GOALS performance scores were significantly higher in the feedback compared to the control group (17.5 ± 4.4 vs. 16.0 ± 3.8, *p* < 0.001; 6.6 ± 2.3 vs. 5.9 ± 2.1, *p* = 0.001, respectively). When comparing total global (26.5 ± 7.3 vs. 23.4 ± 5.1, *p* = 0.003) and task-specific (47.6 ± 12.9 vs. 36.0 ± 12.8, *p* < 0.001) OSATS performance scores for all 4 LCs, the feedback group had performed better than the control group (Table 1).

**Table 1:** Outcome parameters for all laparoscopic cholecystectomies

Parameter	Feedback group	Control group	*p*-value
GOALS score global	17.5 ± 4.4	16.0 ± 3.8	< 0.001
GOALS score task-specific	6.6 ± 2.3	5.9 ± 2.1	0.005
OSATS score global	26.5 ± 7.3	23.4 ± 5.1	0.003
OSATS score task-specific	47.6 ± 12.9	36.0 ± 12.8	< 0.001
CVS achieved (%)	75.2	24.8	< 0.001
Total time (minutes)	84.1 ± 35.9	68.2 ± 32.7	< 0.001
VAS for LC difficulty	33.7 ± 9.2	29.7 ± 8.4	< 0.001
Complication rate	2.9 ± 2.1	3.7 ± 2.3	0.001

Data are presented as number (percentage) for categorical variables, mean standard deviation ± for normally distributed or median, and [25th and 75th percentile] for not normally distributed continuous variables. Accordingly, Chi-Quadrat, exact Fisher, Student’s t-test, or Mann–Whitney *U* test was used to compare

*GOALS* global operative assessment of laparoscopic skills; *OSATS* objective structured assessments of technical skills; *VAS* visual assessment scale; *LC* laparoscopic cholecystectomy

Other than task-specific OSATS, all performance scores of the first LC were comparable between the two groups (Table 2). The performance improvement according to the global and task-specific GOALS and OSATS scores was observed after the second LC (Table 2).

**Table 2:** Comparison of performance scores for individual laparoscopic cholecystectomies

	Feedback group	Control group	*p*-value
LC 1			
GOALS score global	13.4 ± 3.4	14.2 ± 3	0.253
GOALS score task-specific	4.9 ± 2.7	5.0 ± 2.2	0.785
OSATS score global	19.4 ± 5.1	20.1 ± 4.5	0.503
OSATS score task-specific	39 ± 10.3	32 ± 12	0.006
LC 2			
GOALS score global	17.2 ± 3.7	15.1 ± 4	0.020
GOALS score task-specific	6.5 ± 2.2	5.3 ± 2.2	0.014
OSATS score global	25.6 ± 8.4	22.8 ± 4.9	0.068
OSATS score task-specific	44.6 ± 10.8	36.2 ± 13.4	0.003
LC 3			
GOALS score global	19.4 ± 3.8	16.7 ± 3.2	0.001
GOALS score task-specific	7.2 ± 1.6	6.6 ± 1.5	0.065
OSATS score global	28.4 ± 6.2	24.8 ± 4.1	0.003
OSATS score task-specific	52.1 ± 12.4	37.4 ± 11.2	< 0.001
LC 4			
GOALS score global	20.2 ± 3.3	17.9 ± 3.9	0.005
GOALS score task-specific	7.7 ± 1.4	6.7 ± 1.7	0.006
OSATS score global	28.8 ± 4.8	26 ± 4.9	0.012
OSATS score task-specific	54.7 ± 12.1	38.3 ± 13.8	< 0.001

Data are presented as number (percentage) for categorical variables, mean standard deviation ± for normally distributed or median, and [25th and 75th percentile] for not normally distributed continuous variables. Accordingly, Chi-Quadrat, exact Fisher, Student’s t-test, or Mann–Whitney *U* test was used to compare

*LC* laparoscopic cholecystectomy; *GOALS* global operative assessment of laparoscopic skills; *OSATS* objective structured assessments of technical skills

### Secondary outcomes

**Fig. 4:**
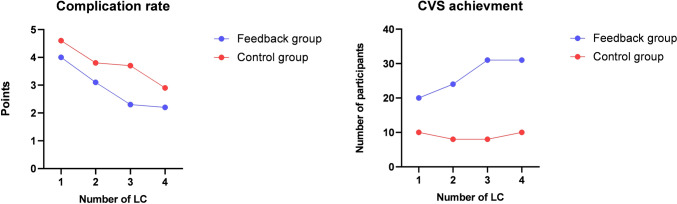
Comparison of complication rate and CVS achievement of the performed LCs between the feedback and the control group

The feedback group achieved CVS more often than the control group (75.2% vs. 24.8%, *p* < 0.001) (Table 1) (Fig. 4). Despite having to perform more difficult LCs compared to the control group (33.7 ± 9.2 vs. 29.7 ± 8.4, *p* < 0.001), the feedback group had fewer complications than the control group (2.9 ± 2.1 vs. 3.7 ± 2.3, *p* = 0.001) (Table 1) (Fig. 4).

The total time of the performed LCs was significantly shorter in the control than in the feedback group (68.2 ± 32.7 vs. 84.1 ± 35.9 min, *p* < 0.001) (Table 1).

**Fig. 5:**
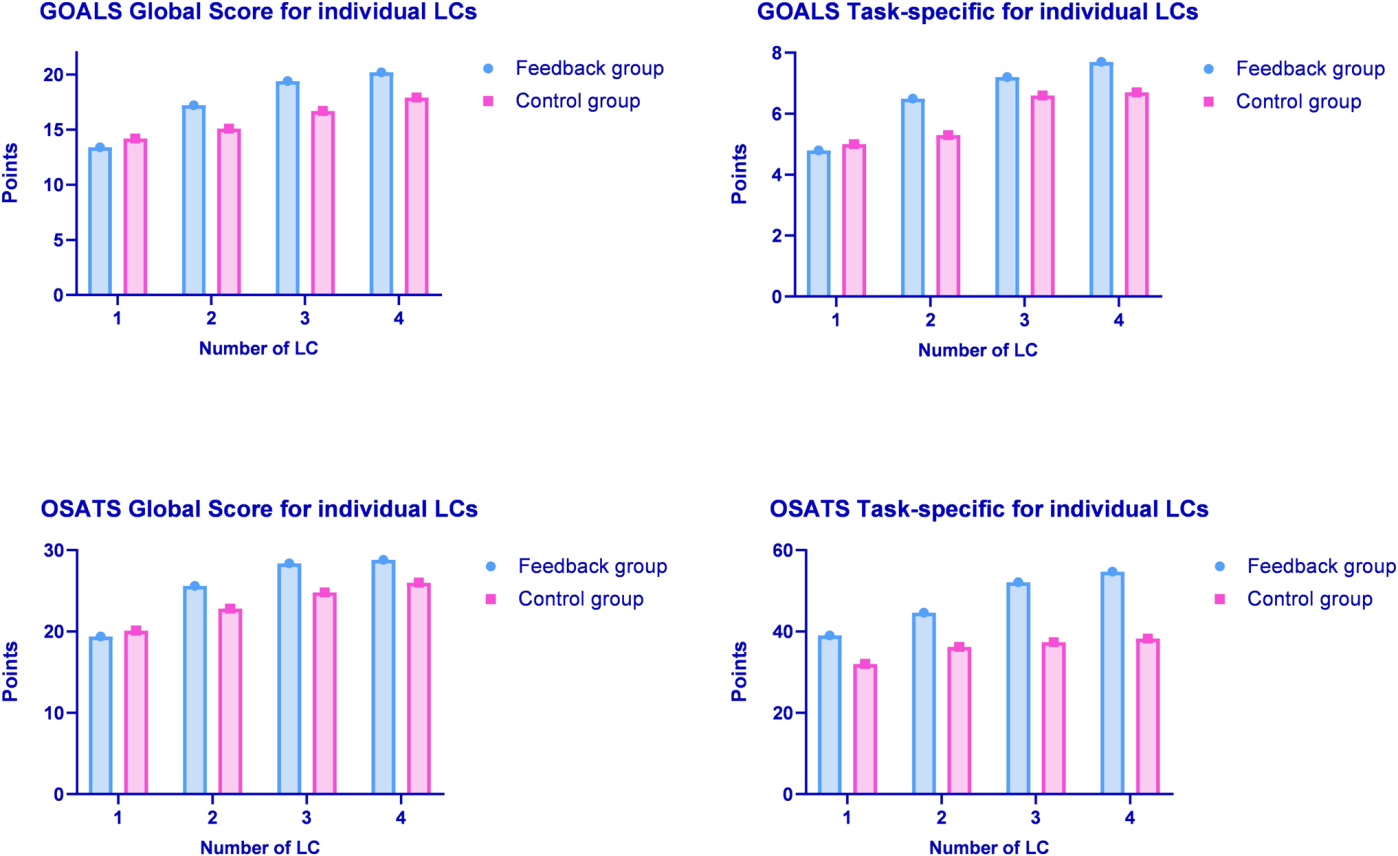
Comparison of the global and task-specific GOALS and OSATS performance scores for each LC between the feedback and the control group

When comparing global and task-specific GOALS and OSATS performance scores for each LC individually, the feedback group showed continuous performance improvement with comparable baseline scores after the first LC (Fig. 5).

**Fig. 6:**
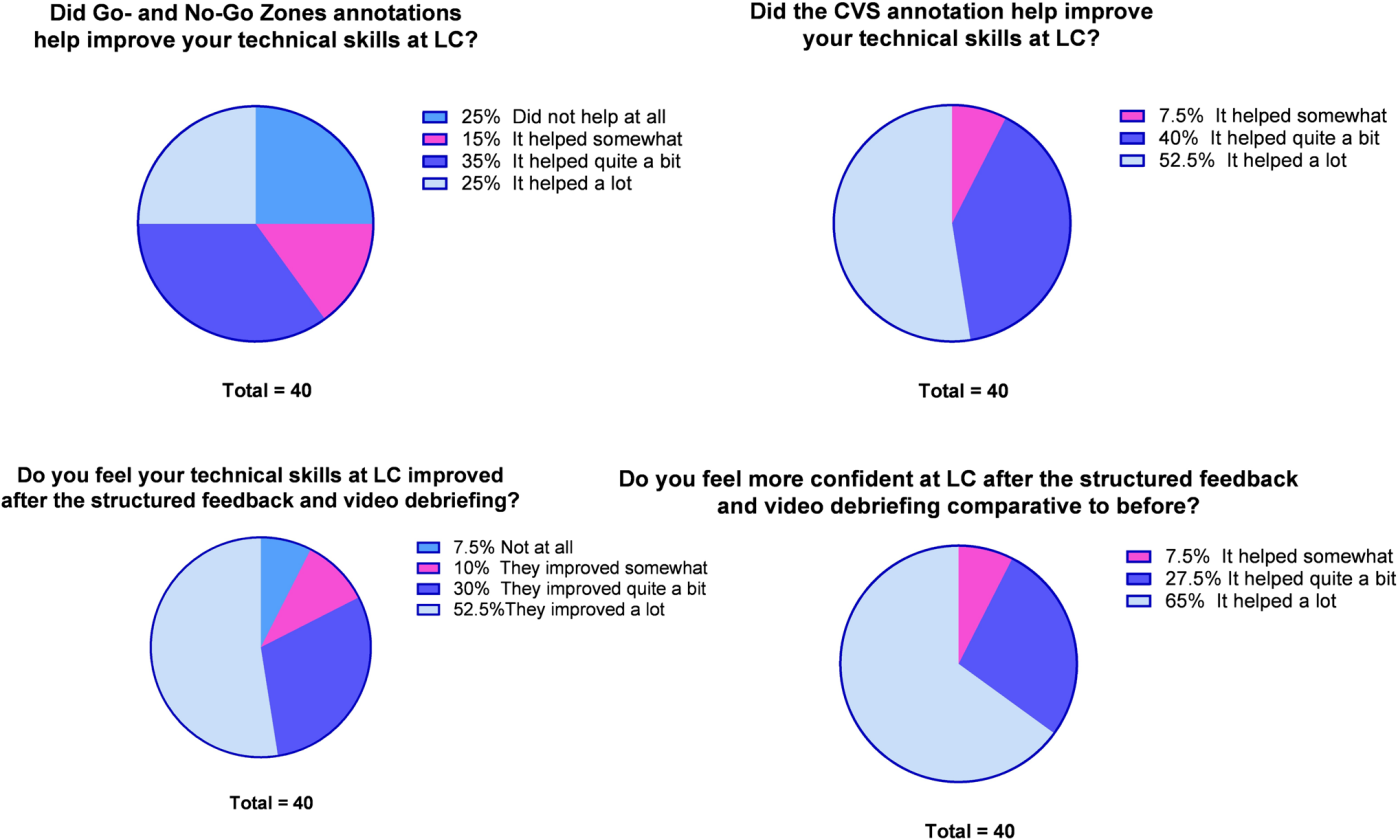
Questionnaire about the subjective effects of the annotations of Go- and No-Go zones and CVS, and structured feedback and video debriefing after each LC on improving performance, confidence, and technical skills during LC in the feedback group

After completing the training, the feedback group was asked to fill out a predefined questionnaire regarding the subjective effects of the training. The annotations of the Go- and No-Go zones were perceived to have helped improve the technical skills of LC in 75% of the students. Fifty-two percent of the trainees found the annotation of the CVS very helpful for their overall operative performance. The structured feedback and video debriefing with CVS annotation made 65% of the trainees feel more confident performing an LC than before (Fig. 6).

## Discussion

This randomized-controlled single-center study showed that structured feedback and video debriefing with CVS annotations in teams of two after each LC (*n* = 4) contributed to the performance improvement according to global and task-specific GOALS and OSATS performance scores. The initial scores after the first LC, except for task-specific OSATS scores, were comparable between the two groups. There is no objective reason why the task-specific OSATS score would differ between the groups since the groups consisted of trainees of comparable previous training but with no previous MIS experience. Nonetheless, the tendency for performance improvement over time was significantly more visible in the feedback than in the control group, which shows the positive continuity of learning effects of structured feedback and video debriefing with CVS annotations on LC performance.

To ensure safe and successful outcomes in MIS, surgeons should participate in structured, comprehensive MIS training programs to develop skills in precise instrument handling, hand–eye coordination, and effective teamwork [40–44]. Individual feedback and video debriefing have positively affected MIS training [21–26, 45]. These days, several online platforms allow surgeons to upload their surgical procedures and analyze or receive feedback or video debriefing from their peers to improve their performance (https://www.csats.com/, https://medtube.net/) [46]. However, there has not been an active incorporation of the CVS annotation as an important safety step in LC as well as the segmentation of the safe (Go) and dangerous (No-Go) zones of dissection into the video debriefing and structured feedback [28, 47–50]. These intervention aspects make this study unique in its design. Furthermore, the intervention was performed on ex-vivo porcine LC, the next closest LC training form to the real LC, enabling a realistic and safe space for practicing and improving surgical skills. The study emphasized that the time invested in the annotation/segmentation aspect of the intervention is worthwhile to improve surgical skills and reduce complications in a safe training setting.

A study by O'Connell et al. evaluated targeted video feedback on LC performed by individual trainees [23]. The video feedback positively impacted the performance with an improved demonstration of CVS and the dissection of Calot’s triangle. Similarly, the feedback group in the present study had significantly higher global and task-specific GOALS and OSATS scores in total and after each individual postinterventional LC compared to the control group. Implementing structured feedback and video debriefing containing CVS annotation in the surgical training of young surgical residents could shorten the LC learning curve and improve performance in LC, a standard training procedure in most hospitals [51].

The reason for choosing the training in teams of two was a study by Kowalewski et al., which evaluated the performance improvement of trainees in teams of two compared to individual trainees undergoing laparoscopic training courses [39]. It showed reduced operation time in the group where trainees were in teams of two and presented the training model as a promising alternative when training time and resources are limited. However, although both groups were trained in teams of two, performance improvement, according to the objective assessment through global and task-specific GOALS and OSATS scores, was observed more in the feedback than in the control group.

Video debriefing after surgery also reduces technical errors in MIS [45]. Hamad et al. reported a significantly reduced technical error rate in laparoscopic jejunojejunal anastomosis in surgical residents who received postoperative video debriefing compared to those who did not receive the postoperative video debriefing [45]. Similarly, the present study's feedback group experienced performance improvement and had fewer complications after structured feedback and video debriefing with CVS annotation. A reduction in complication rates was reached despite the greater difficulty of LCs in the feedback group. This indicates that structured feedback and video debriefing with CVS annotation can be a valuable training modality for LC training.

Achieving CVS is essential to perform a safe LC [49, 50, 52, 53]. Inadequate completion of CVS during LC has been identified as one of the contributory factors of common biliary duct injury [53]. An intraoperative 5-s-long time-out to verify CVS has been shown to improve the CVS achievement rate and, therefore, can assist in avoiding typical intraoperative pitfalls [28]. The feedback group in the presented study performed better and achieved CVS more often than the control group, which is essential in performing a safe LC in a real OR setting [49, 52, 54]. However, the feedback group had an initial higher CVS achievement than the control group even before the intervention, which could potentially influence the CVS achievement discrepancy. Nevertheless, the improvement tendency of CVS achievement in the feedback group was observed over time compared to the flattened CVS achievement curve in the control group. The low rate of CVS achievement in the control group could be attributed to a lack of active and structured feedback on individual steps of LC and emphasizing errors from the previous LCs. Due to the non-existent postprocedural error aspect and reflection, the control group failed to improve the initial low CVS achievement rate. This further emphasizes that novice surgeons need postprocedural feedback reflecting on the potential improvement of the technical steps at the beginning of their training.

In this study, we also observed that the achievement rate of the CVS remained low in both groups. A possible reason for this could be the fact that the trainees had no previous surgical experience. The trainees were at the beginning of their MIS learning curve, and further improvements are expected as their experience increases over time [55]. The second reason could be the thinner porcine anatomical CVS structures, which makes CVS recognition and achievement more difficult.

It was interesting to observe that the students from the control group were faster in total operative time than those from the feedback group. The prolonged operative time in the feedback group may result from careful attention to the important anatomical structures and safety of the procedure since the feedback group had significantly fewer complications and achieved CVS more often than the control group. Although prolonged operative time can be associated with an increased risk of postoperative complications such as wound infections and pneumonia, it should not be the only factor for surgical quality assessment and clinical outcome prediction. Operative time has been criticized as a performance measure because it does not necessarily reflect the quality of the performance [56–60].

Madani et al. described the potential of AI-based intraoperative guidance through semantic segmentation with the identification of safe (Go) and dangerous (No-Go) zones of dissection during LC [29] in order to achieve safer LC performance and reduce intraoperative complications [61]. In the present study, a similar concept of annotating the Go and No-Go zones of dissection has been used as part of the structured feedback and video debriefing with a CVS annotation after each LC, which led to a significant performance improvement and reduced intraoperative complication rate. As a byproduct, these annotations can be used for Surgical Science Data (SDS) research and development projects, and can thus create synergies between training, research, and development in surgery [62].

Most trainees reported that structured feedback and video debriefing of Go- and No-Go-Zones and CVS annotations helped them improve their technical skills and confidence in performing LC. This aspect of the training is valuable since the sense of confidence and performance are mutually corresponding and can reduce subjective workload in MIS [63, 64].

MIS training offers many modalities that are often combined, such as blended learning [7], box-trainer, VR trainer [6, 9], augmented reality [14], telementoring [65], and telestration [17, 19]. Combining these advantageous modalities with other beneficial factors, such as training in teams of two and postprocedural feedback and video debriefing with CVS annotation, could improve MIS training [39, 66, 67].

There are some limitations to be addressed regarding the present study. The participants of the study were medical students without previous MIS experience. This limits the transfer of the study results into a clinical setting because surgical residents would have different surgical predispositions. Despite randomizing trainees without previous surgical experience, the feedback group had increased initial CVS achievement and task-specific OSATS score after the first LC, even before structured feedback and video debriefing with CVS annotation took place. However, the progressive continuity of the observed learning curve regarding performance and CVS achievement demonstrated positive effects of the intervention over time and repetitions. Other scores, such as global and task-specific GOALS and global OSATS, were comparable after the first LC. The preclinical setting of the study could be seen as a potential limitation, and the concept of it needs to be tested in a clinical setting to verify the results. Lastly, the intervention contained three potentially independent factors (structured feedback, video debriefing and annotation), which could have been applied individually. This makes it impossible to discern the impact of each intervention individually.

The study assessed the effects of structured feedback and video debriefing with CVS annotation on ex-vivo porcine LC performance compared to verbal instructions on demand only. That is why there is no comparison between the effects of structured feedback and video debriefing and CVS annotation with other training modalities, such as image-guided surgery or telestration with augmented reality. This should be a subject of future randomized-controlled studies to optimize MIS training.

The present randomized-controlled study showed a positive impact of structured feedback and video debriefing with CVS annotation after ex-vivo porcine LC in novice trainees. Achievement of CVS was improved, and complication rates were reduced. The structured feedback and video debriefing also contributed to increased self-confidence and subjective safety of the procedure. This is one of the reasons why postoperative structured feedback and video debriefing with CVS annotation could thus be integrated into preclinical and clinical training. In the near future, automated intraoperative feedback with artificial intelligence models, such as assessment of CVS achievement [36] and intraoperative image guidance like Go- and No-Go-Zones definition [29], will become available in daily routine. A reflection on the performed surgery and aspiration for continuous improvement through structured feedback and video debriefing with an experienced and skilled mentor should always form the basis for continuous improvement of surgical quality and skills.

## References

[1] Qu H, Liu Y, He QS (2014). Short- and long-term results of laparoscopic versus open anti-reflux surgery: a systematic review and meta-analysis of randomized controlled trials. J Gastrointest Surg.

[2] Agha R, Muir G (2003). Does laparoscopic surgery spell the end of the open surgeon?. J R Soc Med.

[3] Pucher PH, Sodergren MH, Singh P, Darzi A, Parakseva P (2013). Have we learned from lessons of the past? A systematic review of training for single incision laparoscopic surgery. Surg Endosc.

[4] Hung AJ, Chen J, Shah A, Gill IS (2018). Telementoring and telesurgery for minimally invasive procedures. J Urol.

[5] Liceaga A, Fernandes LF, Romeo A, Gagstatter F (2013). Romeo's gladiator rule: knots, stitches and knot tying techniques: a tutorial based on a few simple rules; new concepts to teach suturing techniques in laparoscopic surgery.

[6] Kowalewski KF, Garrow CR, Proctor T, Preukschas AA, Friedrich M, Müller PC (2018). LapTrain: multi-modality training curriculum for laparoscopic cholecystectomy—results of a randomized controlled trial. Surg Endosc.

[7] Schmidt MW, Kowalewski KF, Trent SM, Benner L, Müller-Stich BP, Nickel F (2020). Self-directed training with e-learning using the first-person perspective for laparoscopic suturing and knot tying: a randomised controlled trial: learning from the surgeon's real perspective. Surg Endosc.

[8] Yu P, Pan J, Wang Z, Shen Y, Li J, Hao A (2022). Quantitative influence and performance analysis of virtual reality laparoscopic surgical training system. BMC Med Educ.

[9] Nickel F, Brzoska JA, Gondan M, Rangnick HM, Chu J, Kenngott HG (2015). Virtual reality training versus blended learning of laparoscopic cholecystectomy: a randomized controlled trial with laparoscopic novices. Medicine.

[10] Kowalewski KF, Hendrie JD, Schmidt MW, Garrow CR, Bruckner T, Proctor T (2017). Development and validation of a sensor- and expert model-based training system for laparoscopic surgery: the iSurgeon. Surg Endosc.

[11] Yiannakopoulou E, Nikiteas N, Perrea D, Tsigris C (2015). Virtual reality simulators and training in laparoscopic surgery. Int J Surg.

[12] Snyderman CH, Gardner PA, Lanisnik B, Ravnik J (2016). Surgical telementoring: a new model for surgical training. Laryngoscope.

[13] Kowalewski K-F, Hendrie JD, Schmidt MW, Proctor T, Paul S, Garrow CR (2017). Validation of the mobile serious game application Touch Surgery™ for cognitive training and assessment of laparoscopic cholecystectomy. Surg Endosc.

[14] Luck J, Hachach-Haram N, Greenfield M, Smith O, Billingsley M, Heyes R (2017). Augmented reality in undergraduate surgical training: the PROXIMIE pilot. Int J Surg.

[15] Roch PJ, Rangnick HM, Brzoska JA, Benner L, Kowalewski KF, Muller PC (2018). Impact of visual-spatial ability on laparoscopic camera navigation training. Surg Endosc.

[16] Vajsbaher T, Ziemer T, Schultheis H (2020). A multi-modal approach to cognitive training and assistance in minimally invasive surgery. Cogn Syst Res.

[17] Felinska EA, Fuchs TE, Kogkas A, Chen ZW, Otto B, Kowalewski KF (2023). Telestration with augmented reality improves surgical performance through gaze guidance. Surg Endosc.

[18] Kowalewski KF, Garrow CR, Schmidt MW, Benner L, Muller-Stich BP, Nickel F (2019). Sensor-based machine learning for workflow detection and as key to detect expert level in laparoscopic suturing and knot-tying. Surg Endosc.

[19] Wild C, Lang F, Gerhäuser AS, Schmidt MW, Kowalewski KF, Petersen J (2022). Telestration with augmented reality for visual presentation of intraoperative target structures in minimally invasive surgery: a randomized controlled study. Surg Endosc.

[20] Nickel F, Hendrie JD, Kowalewski K-F, Bruckner T, Garrow CR, Mantel M (2016). Sequential learning of psychomotor and visuospatial skills for laparoscopic suturing and knot tying—a randomized controlled trial “The Shoebox Study” DRKS00008668. Langenbecks Arch Surg.

[21] Ahlborg L, Weurlander M, Hedman L, Nisel H, Lindqvist PG, Felländer-Tsai L (2015). Individualized feedback during simulated laparoscopic training:a mixed methods study. Int J Med Educ.

[22] Atkinson A, Watling CJ, Brand PLP (2022). Feedback and coaching. Eur J Pediatr.

[23] O'Connell L, McKevitt K, Khan W, Waldron R, Khan I, Barry K (2020). Impact of targeted trainer feedback via video review on trainee performance of laparoscopic cholecystectomy. Surgeon.

[24] Ghaderi I, Tran T, Carton M, Samamé J, Galvani C (2021). The impact of intensive laparoscopic training course with structured assessment and immediate feedback on residents' operative performance in animal lab. Surg Endosc.

[25] Sterz J, Linßen S, Stefanescu MC, Schreckenbach T, Seifert LB, Ruesseler M (2021). Implementation of written structured feedback into a surgical OSCE. BMC Med Educ.

[26] Hira S, Singh D, Kim TS, Gupta S, Hager G, Sikder S (2022). Video-based assessment of intraoperative surgical skill. Int J Comput Assist Radiol Surg.

[27] Singh P, Aggarwal R, Tahir M, Pucher PH, Darzi A (2015). A Randomized controlled study to evaluate the role of video-based coaching in training laparoscopic skills. Ann Surg.

[28] Mascagni P, Rodríguez-Luna MR, Urade T, Felli E, Pessaux P, Mutter D (2021). Intraoperative time-out to promote the implementation of the critical view of safety in laparoscopic cholecystectomy: a video-based assessment of 343 procedures. J Am Coll Surg.

[29] Madani A, Namazi B, Altieri MS, Hashimoto DA, Rivera AM, Pucher PH (2022). Artificial intelligence for intraoperative guidance: using semantic segmentation to identify surgical anatomy during laparoscopic cholecystectomy. Ann Surg.

[30] Moher D, Hopewell S, Schulz KF, Montori V, Gøtzsche PC, Devereaux PJ (2012). CONSORT 2010 explanation and elaboration: updated guidelines for reporting parallel group randomised trials. Int J Surg.

[31] Pape-Koehler C, Chmelik C, Aslund AM, Heiss MM (2010). An interactive and multimedia-based manual of surgical procedures: Webop–an approach to improve surgical education. Zentralbl Chir.

[32] Mutter D, Vix M, Dallemagne B, Perretta S, Leroy J, Marescaux J (2011). WeBSurg: an innovative educational Web site in minimally invasive surgery–principles and results. Surg Innov.

[33] Lucas S, Tuncel A, Bensalah K, Zeltser I, Jenkins A, Pearle M (2008). Virtual reality training improves simulated laparoscopic surgery performance in laparoscopy naive medical students. J Endourol.

[34] Hiemstra E, Kolkman W, Wolterbeek R, Trimbos B, Jansen FW (2011). Value of an objective assessment tool in the operating room. Can J Surg.

[35] Wang J, Fan X, Qin S, Shi K, Zhang H, Yu F (2022). Exploration of the efficacy of radiomics applied to left ventricular tomograms obtained from D-SPECT MPI for the auxiliary diagnosis of myocardial ischemia in CAD. Int J Cardiovasc Imaging.

[36] Mascagni P, Vardazaryan A, Alapatt D, Urade T, Emre T, Fiorillo C (2022). Artificial intelligence for surgical safety: automatic assessment of the critical view of safety in laparoscopic cholecystectomy using deep learning. Ann Surg.

[37] Vassiliou MC, Feldman LS, Andrew CG, Bergman S, Leffondré K, Stanbridge D (2005). A global assessment tool for evaluation of intraoperative laparoscopic skills. Am J Surg.

[38] Willuth E, Hardon SF, Lang F, Haney CM, Felinska EA, Kowalewski KF (2021). Robotic-assisted cholecystectomy is superior to laparoscopic cholecystectomy in the initial training for surgical novices in an ex vivo porcine model: a randomized crossover study. Surg Endosc.

[39] Kowalewski K-F, Minassian A, Hendrie JD, Benner L, Preukschas AA, Kenngott HG (2019). One or two trainees per workplace for laparoscopic surgery training courses: results from a randomized controlled trial. Surg Endosc.

[40] de Angelis N, Marchegiani F, Schena CA, Khan J, Agnoletti V, Ansaloni L (2023). Training curriculum in minimally invasive emergency digestive surgery: 2022 WSES position paper. World J Emerg Surg.

[41] Kowalewski KF, Seifert L, Kohlhas L, Schmidt MW, Ali S, Fan C (2023). Video-based training of situation awareness enhances minimally invasive surgical performance: a randomized controlled trial. Surg Endosc.

[42] Yoshida S, Miyano G, Tanaka M, Ikegami M, Kato H, Seo S (2021). Cadaver Training for Minimally Invasive Pediatric Surgery: A Preliminary Report. J Laparoendosc Adv Surg Tech A.

[43] Lehr EJ, Guy TS, Smith RL, Grossi EA, Shemin RJ, Rodriguez E (2016). Minimally invasive mitral valve surgery III: training and robotic-assisted approaches. Innovations (Phila).

[44] Falcioni AG, Yang HC, Maricic MA, Rodriguez SP, Bailez MM (2022). Effectiveness of telesimulation for pediatric minimally invasive surgery essential skills training. J Pediatr Surg.

[45] Hamad GG, Brown MT, Clavijo-Alvarez JA (2007). Postoperative video debriefing reduces technical errors in laparoscopic surgery. Am J Surg.

[46] Ketel MHM, Klarenbeek BR, Eddahchouri Y, Cheong E, Cuesta MA, van Daele E (2023). A video-based procedure-specific competency assessment tool for minimally invasive esophagectomy. JAMA Surg.

[47] Gupta V (2023). How to achieve the critical view of safety for safe laparoscopic cholecystectomy: technical aspects. Ann Hepatobiliary Pancreat Surg.

[48] Jin Y, Liu R, Chen Y, Liu J, Zhao Y, Wei A (2022). Critical view of safety in laparoscopic cholecystectomy: a prospective investigation from both cognitive and executive aspects. Front Surg.

[49] Manatakis DK, Antonopoulou MI, Tasis N, Agalianos C, Tsouknidas I, Korkolis DP (2023). Critical view of safety in laparoscopic cholecystectomy: a systematic review of current evidence and future perspectives. World J Surg.

[50] Yamashita Y, Kimura T, Matsumoto S (2010). A safe laparoscopic cholecystectomy depends upon the establishment of a critical view of safety. Surg Today.

[51] Fahrner R, Turina M, Neuhaus V, Schöb O (2012). Laparoscopic cholecystectomy as a teaching operation: comparison of outcome between residents and attending surgeons in 1,747 patients. Langenbecks Arch Surg.

[52] Sgaramella LI, Gurrado A, Pasculli A, de Angelis N, Memeo R, Prete FP (2021). The critical view of safety during laparoscopic cholecystectomy: strasberg yes or no? An Italian Multicentre study. Surg Endosc.

[53] Way LW, Stewart L, Gantert W, Liu K, Lee CM, Whang K (2003). Causes and prevention of laparoscopic bile duct injuries: analysis of 252 cases from a human factors and cognitive psychology perspective. Ann Surg.

[54] Terho P, Sallinen V, Lampela H, Harju J, Koskenvuo L, Mentula P (2021). The critical view of safety and bile duct injuries in laparoscopic cholecystectomy: a photo evaluation study on 1532 patients. HPB (Oxford).

[55] Hopper AN, Jamison MH, Lewis WG (2007). Learning curves in surgical practice. Postgrad Med J.

[56] Daley BJ, Cecil W, Clarke CP, Cofer JB, Guillamondegui OD (2015). How slow is too slow? Correlation of operative time to complications: an analysis from the Tennessee surgical quality collaborative. J Am Coll Surg.

[57] Procter LD, Davenport DL, Bernard AC, Zwischenberger JB (2010). General surgical operative duration is associated with increased risk-adjusted infectious complication rates and length of hospital stay. J Am Coll Surg.

[58] Ingraham AM, Richards KE, Hall BL, Ko CY (2010). Quality improvement in surgery: the American college of surgeons national surgical quality improvement program approach. Adv Surg.

[59] Ko CY, Martin G, Dixon-Woods M (2022). Three observations for improving efforts in surgical quality improvement. JAMA Surg.

[60] Ko CY, Shah T, Nelson H, Nathens AB (2022). Developing the American college of surgeons quality improvement framework to evaluate local surgical improvement efforts. JAMA Surg.

[61] Laplante S, Namazi B, Kiani P, Hashimoto DA, Alseidi A, Pasten M (2023). Validation of an artificial intelligence platform for the guidance of safe laparoscopic cholecystectomy. Surg Endosc.

[62] Maier-Hein L, Eisenmann M, Sarikaya D, März K, Collins T, Malpani A (2022). Surgical data science - from concepts toward clinical translation. Med Image Anal.

[63] Clanton J, Gardner A, Cheung M, Mellert L, Evancho-Chapman M, George RL (2014). The relationship between confidence and competence in the development of surgical skills. J Surg Educ.

[64] Yurko YY, Scerbo MW, Prabhu AS, Acker CE, Stefanidis D (2010). Higher mental workload is associated with poorer laparoscopic performance as measured by the NASA-TLX Tool. Simul Healthcare.

[65] Forgione A, Kislov V, Guraya SY, Kasakevich E, Pugliese R (2015). Safe introduction of laparoscopic colorectal surgery even in remote areas of the world: the value of a comprehensive telementoring training program. J Laparoendosc Adv Surg Tech A.

[66] Herrera-Almario GE, Kirk K, Guerrero VT, Jeong K, Kim S, Hamad GG (2016). The effect of video review of resident laparoscopic surgical skills measured by self- and external assessment. Am J Surg.

[67] van Dalen A, van Haperen M, Swinkels JA, Grantcharov TP, Schijven MP (2021). Development of a model for video-assisted postoperative team debriefing. J Surg Res.

